# A member of the CAP protein superfamily, *Hc*-CAP-15, is important for the parasitic-stage development of *Haemonchus contortus*

**DOI:** 10.1186/s13071-023-05907-w

**Published:** 2023-08-17

**Authors:** Hui Liu, Zhuolin Tao, Yifan Wang, Xin Liu, Chunqun Wang, Lu Liu, Min Hu

**Affiliations:** State Key Laboratory of Agricultural Microbiology, College of Veterinary Medicine, Huazhong Agricultural University, Wuhan, 430070 China

**Keywords:** *Haemonchus contortus*, *Hc*-CAP-15, RNA interference, Development

## Abstract

**Background:**

The CAP superfamily proteins are distributed widely in eukaryotes and play crucial roles in various biological processes. However, very little is known about their functions in parasitic nematodes, including *Haemonchus contortus*, a socioeconomically important parasitic nematode. We have therefore studied a member of the CAP protein family of *H. contortus*, named *Hc-*CAP-15, with the aim to explore its roles in regulating the parasitic developmental process.

**Methods:**

The conservation and phylogenetic relationships, spatial expression and temporal transcription profiles of *Hc*-CAP/*cap*-*15*, as well its biological function during parasite development were investigated using bioinformatics, immunofluorescence, real-time PCR and RNA interference (RNAi).

**Results:**

*Hc*-CAP-15 was found to be a single-domain CAP protein consisting of four conserved motifs that is localized in the cuticle, intestine and oocyte of adult worms. *Hc-cap-15* was transcribed at all developmental stages of *H. contortus*, with the highest transcription level in parasitic fourth-stage larvae (L4s). Silencing of *Hc-cap-15* resulted in a significant increase in the body length of L4s.

**Conclusions:**

The results suggested that *Hc*-CAP-15 is important for the development of *H. contortus*. Our findings provide a basis for further study of the functions of the CAP family proteins in *H. contortus* and related parasitic nematodes.

**Graphical Abstract:**

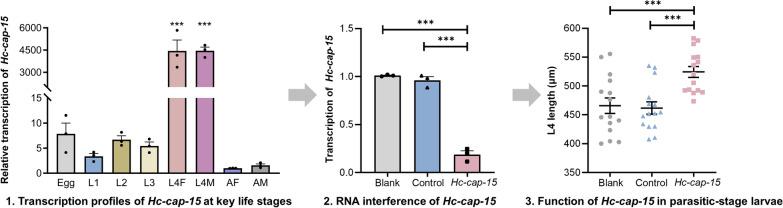

**Supplementary Information:**

The online version contains supplementary material available at 10.1186/s13071-023-05907-w.

## Background

Nematoda is one of the largest phyla in the animal kingdom, with more than 28,000 recorded species of nematodes, of which nearly 50% (about 16,000 species) are parasitic [1]. Parasitic nematodes are important pathogens that cause debilitating chronic infections in humans and animals, resulting in a serious disease burden worldwide [2–5]. In addition, the economic consequences of infections caused by nematodes to economically significant animals was estimated at $US30 billion globally [6, 7]. Nowadays, the treatment of nematode diseases relies heavily on a restricted number of anthelmintics, which has led to the emergence and spread of drug resistance [8, 9]. The rapid expansion of anthelmintic resistance has dramatically increased the need for alternative and sustainable control strategies. To develop such new interventions, an improved understanding of the molecular biology of these pathogens is a priority.

The CAP (cysteine-rich secretory proteins, antigen 5 and pathogenesis-related 1) superfamily of proteins, also known as SCPs/ASPs/VALs, is characterized by the presence of an approximately 15-kDa cysteine-rich CAP domain composed of one to four CAP motifs (InterProScan No. IPR01404) [10]. Members of the CAP protein superfamily are widely distributed among helminth species [11, 12], and a total of 3915 putative encoding genes have been recorded to be present in the genome dataset of 81 major parasitic worms [13]. Furthermore, CAP protein expression has been found to be significantly upregulated at the parasitic stage compared with the free-living stage, as previously described in *Haemonchus contortus* [14], *Ancylostoma caninum* [15], *Cooperia oncophora* and *Ostertagia ostertagi* [16]. A typical example was shown in a *Strongyloides* nematode, where a large number of CAP encoding genes were identified in the parasitic stage of nematodes [17]. CAP proteins have also been found as a prominent component of secreted products in parasitic-stage nematodes (e.g. *H. contortus* [18], *Heligmosomoides polygyrus* [19] and *Nippostrongylus brasiliensis* [20]), which suggests that these proteins could play a vital role in regulating the development of parasitic-stage worms or host–parasite interactions. Given these properties, CAP proteins are being investigated as potential vaccine candidates against human or animal parasitic nematode diseases [21–24]. Although considered crucial for the fundamental biological processes in nematodes [25], little is known about the function of CAP proteins in regulating nematode development.

*Haemonchus contortus* is a highly pathogenic blood-feeding gastrointestinal nematode of ruminants that causes anemia and even death [26]. As the global goat stock increases, the prevalence of haemonchosis is also increasing, causing severe losses to the livestock industry worldwide [27]. Genomic data on *H. contortus* shows that there are 237 putative CAP-encoding genes [28]. Despite this extremely high abundance, there is a paucity of understanding about the function of these molecules in *H. contortus*. Clarifying the biological significance of CAP proteins will provide a solid basis for developing effective and sustainable interventions for haemonchosis. Here, we selected *Hc-cap-15*, which has the highest transcriptional reads in parasitic fourth-stage larvae (L4s) based on the transcriptional data of CAP proteins of *H. contortus* [29], and used bioinformatics, immunofluorescence, real-time PCR and RNA interference (RNAi) to initially investigate the expression and function of *Hc-cap-15*-encoding protein.

## Methods

### Parasite materials

Healthy Boer goats aged 3–4 months were orally infected with 7000 infective third-stage larvae (iL3s; Haecon-5 strain) and maintained in a nematode-free environment. Eggs were isolated from feces using the saturated sucrose flotation method on day 21 post-infection. The collected eggs were incubated in a cell culture flask at 25 °C containing Earle’s balanced salt solution (EBSS; Sigma-Aldrich, St. Louis, MO, USA) supplemented with 0.5% (w/v) of yeast extract to gather the first- (L1s), second- (L2s), and third-stage larvae (L3s) on days 1, 4 and 7, respectively [30]. The L4s and adults were obtained from the abomasum of goats infected on day 9 and day 21 post-challenge, respectively. All parasite materials were washed with sterile phosphate-buffered saline (PBS) and stored in liquid nitrogen until use.

### Bioinformatic analyses

BlastP of the National Center for Biotechnology Information (NCBI) (http://www.ncbi.nlm.nih.gov/BLAST) was used to search for homologous sequences of *Hc*-CAP-15 (GenBank: ALA23470.1). Multiple sequence alignments were performed using the CLC Genomics Workbench 23 with default settings (Qiagen, Hilden, Germany). Protein motifs were identified by scanning the InterPro database (https://www.ebi.ac.uk/interpro/) and highlighted using Adobe Illustrator (v.2019; Adobe, San Jose, CA, USA). Phylogenetic analysis of *Hc*-CAP-15 was conducted using MEGA v.7.0 [31]. In brief, homologous sequences of *Hc-*CAP-15 from 12 nematode species were retrieved from NCBI (sequences as shown in Additional file 1: Table S1), and the GLIPR1 protein (a member of the mammalian CAP protein family) of *Urocitellus parryii* was set as an outgroup. The phylogenetic relationship was analyzed using the neighbor-joining (NJ) method, and the confidence limits were assessed using a bootstrap procedure employing 1000 pseudo-replicates.

### Expression of recombinant protein and preparation of anti*-*r*Hc*-CAP-15 polyclonal antibody

The truncated *Hc*-CAP-15 encoding sequence (21–297 amino acids, not containing signal peptide) was amplified by PCR using primer pair r*Hc*-CAP-15-F/R (Additional file 1: Table S1) and cloned into the prokaryotic expression vector *pE*-SUMO using the ClonExpress II One Step Cloning Kit (Vazyme, Nanjing, China). The cycling regimen was: 95 °C for 5 min, followed by 35 cycles at 95 °C for 30 s, 60 °C for 15 s and 72 °C for 1 min. After verification of the sequence, the recombinant expression plasmid was transferred into *Escherichia coli* BL21 (DE3) cells, and the recombinant protein was induced to express using 1 mM isopropyl β-D-1-thiogalactopyranoside (IPTG) for 5 h at 37 °C. The induction of recombinant protein was verified by 12% sodium dodecyl sulfate–polyacrylamide gel electrophoresis (SDS-PAGE). The inclusion body was then resuspended, washed and dissolved in 8 M urea solution; the r*Hc-*CAP-15 protein was then purified by affinity chromatography by passage through a Ni Sepharose 6FF matrix (GE Healthcare, Pittsburgh, IL, USA), and refolding occurred at different concentrations of urea solution.

The r*Hc-*CAP-15 protein was used to immunize rabbits to obtain polyclonal antibodies. In short, two healthy rabbits weighing 3 kg were immunized by subcutaneous injection at multiple points with 500 μg (1 mg/ml, 0.5 ml) of recombinant protein mixed with an equal volume of adjuvant (Freund’s complete adjuvant was used for the first immunization, and Freund’s incomplete adjuvant was used for the subsequent three immunizations) every 2 weeks for a total of four injections. Serum samples were collected before euthanasia of rabbits with pentobarbital, and immunoglobulin G (IgG) antibodies were purified using the protein A + G Agarose Fast Flow chromatography medium (Beyotime, Shanghai, China). The titer of polyclonal antibodies were determined by an enzyme-linked immunosorbent assay (ELISA). In addition, purified polyclonal antibodies were collected before immunization as the negative control. All antibodies were stored at − 20 °C until use.

### Immunofluorescence assay to evaluate the spatial expression of *Hc*-CAP-15

Anti-r*Hc*-CAP-15 antibody was used for the immunoblotting and immunofluorescence studies. For the western blot, the antibody was used to determine whether the native *Hc*-CAP-15 exists in the total protein of adult worms*.* Briefly, 10 adult worms were placed in 1 ml lysis buffer (Beyotime) and homogenized in a glass homogenizer for 30 min on ice for complete lysis of total proteins, followed by centrifugation of the homogenate at 10,000 *g*, 4 °C for 15 min to obtain the protein supernatant. The total natural protein sample was then electrophoresed in 12% SDS-PAGE gels and the products transferred to a polyvinylidene difluoride (PVDF) membrane (Merck KGaA, Darmstadt, Germany). After being blocked with 2% (w/v) bovine serum albumin (BSA) (Beyotime) in PBS containing 20% Tween-20 (PBST) for 2 h at 37 °C, the membrane was incubated with 1:1000 diluted anti-r*Hc*-CAP-15 or with negative antibody overnight at 4 °C. The membrane was then washed 6 times, each wash for 5 min, in PBST and subsequently incubated with the goat anti-rabbit IgG antibody conjugated with horse radish peroxidase (Abbkine, Wuhan, China) diluted 1:3000 in PBST, for 2 h at room temperature. After 6 washes, immunodetection was carried out using by ECL chemiluminescence (Vazyme, Nanjing, China) following the manufacturer’s instructions, and the image was generated through a luminous imaging system (Tanon 5200; Tanon, Shanghai, China).

For the immunofluorescence analysis, the positive antibody was used to determine the location of the protein in adult *H. contortus.* In brief, fresh adult female and male worms obtained from goat abomasum were washed with PBS and fixed with 4% paraformaldehyde at room temperature for 12 h, and then dehydrated through an ethanol solution gradient. The adult female and male worms were then embedded in wax blocks and each block cut longitudinally into 4-μm slices. The slices were dried in an oven at 65 °C for 2 h, dewaxed to water, washed 3 times with PBS, 5 min each time, and then blocked with 5% BSA for 20 min at 37 °C. The sections were incubated with anti-r*Hc*-CAP-15 IgG (1:500) or negative IgG (1:500) at 4 °C overnight. After washing with PBS three times, the sections were subjected to incubation with the Alexa Fluor® 488 goat anti-rabbit IgG antibody ReadyProbes® reagent (Thermo Fisher Scientific, Waltham, MA, USA) at a 1:3000 dilution for 50 min at 37 °C. Slices were washed as described above and then incubated at 24 °C for 5 min with 4,6-diamidino-2-phenylindole (DAPI) solution in the dark, following which the slices were scanned again after a wash (3DHISTECH Ltd., Budapest, Hungary). Images were captured and processed using Caseviewer 2.0 (3DHISTECH Ltd.).

### Assessing transcript abundance of *Hc-cap-15* using real-time PCR

The transcriptional levels of *Hc-cap*-*15* during the entire developmental period of *H. contortus*, including eggs, L1s, L2s, L3s, female L4s (L4F) and male L4s (L4M), female adults (AF) and male adults (AM), were determined by real-time PCR. Briefly, total RNA was extracted from the samples at each developmental period (40,000 eggs, 20,000 L1s and L2s, 10,000 L3s, 5 L4s, and 2 adults of each sex) by using TRIzol reagent (Invitrogen, Thermo Fisher Scientific) following the manufacturer’s protocol, and then complimentary DNA (cDNA) was reverse transcribed using the HiScript II Q RT SuperMix (Vazyme). Real-time PCR was carried out using 2× SYBR Green Master Mix (Takara, Shiga, Japan), and the mean threshold cycle (Ct) values of *Hc-cap*-*15* were determined with specific primer pair q*Hc-cap*-*15*-F/R (Additional file 1: Table S2) in a thermal cycler (model ViiA; Bio-Rad Laboratories, Hercules, CA, USA). *Hc-β-tubulin* was set as the internal reference gene. The cycling protocol comprised 95 °C for 30 s, followed by 35 cycles at 95 °C for 15 s, 60 °C for 15 s and 72 °C for 20 s. The 2^−ΔΔCt^ method was applied to calculate the relative transcription level (AF = 1) at the different developmental stages of *H. contortus* [32].

### RNA interference in vitro

Gene knockdown of *Hc-cap*-*15* was conducted in *H. contortus* using a soaking method as described previously [33]. In brief, according to the coding sequence of *Hc*-*cap*-*15*, three specific small interfering RNA (siRNA) sequences and one negative siRNA sequence (no identity to any *H. contortus* nucleotide sequence) were designed with online software siDirect 2.0 (http://sidirect2.rnai.jp/) and synthesized by GenePharma Co. Ltd. (Shanghai, China) (Additional file 1: Table S3). The siRNAs were diluted to 50 μM with diethylpyrocarbonate (DEPC) water and stored at − 80 ºC until use. Active iL3s were exsheathed with 0.15% sodium hypochlorite solution at 37 ℃ for 10 min, and their morphology was observed under the microscope until 90% of them were exsheathed. Exsheathed L3s (xL3s) were washed 3 times with sterile PBS and EBSS containing penicillin/streptomycin/fungizone (Beyotime). Larvae were incubated in 80 μl of EBSS containing penicillin/streptomycin/fungizone at a concentration of 125,000 xL3s/ml. A 6-μl aliquot of siRNA was first pre-incubated with 5 μl of lipofectamine 2000 (Invitrogen, Thermo Fisher Scientific), 0.5 μl of RNasin (Promega, Madison, WI, USA) and 8.5 μl of EBSS for 15 min at room temperature and then added to xL3s to a final volume of 100 μl. Blanks and controls were used in the assay, with blanks containing 6 μl of EBSS and controls containing 6 μl of negative control siRNA. Larvae were incubated for 72 h at 37 °C under 20% (v/v) CO_2_. Three days later, about 200 larvae were transferred to fresh EBSS medium for further 4 days to assess the developmental level and to measure the body size. The remaining larvae were extracted with total RNA as described above following 6 washes with DEPC water in a glass tube. Transcript levels of *Hc-cap-15* across the different groups were determined by real-time PCR using specific primers (Additional file 1: Table S2) with *Hc*-*β-tubulin* selected as an internal control.

### Determination of L4s developmental rate and measurement of body width and length

The development of L3s to L4s was characterized morphologically based on the presence of a buccal capsule, as described previously [34]. Therefore, the development rate of L4s in vitro was assessed on day 7 after RNAi, and body size of individual L4s was also determined on the same day. In brief, the distance from the head to the tail was considered to be the body length of L4s, and the width of the pharynx was defined as the body width of L4s. All morphological observations described were performed under an Olympus inverted microscope (model IX51; Olympus, Tokyo, Japan). Image acquisition and larval size measurements were conducted using CellSens Standard software (Olympus).

### Statistical analysis

Statistical analysis was conducted using GraphPad Prism 8.0 software (GraphPad Software, San Diego, CA, USA). All data were presented as mean ± standard error of the mean (SEM). One-way analysis of variance (ANOVA) was applied to compare the statistical differences between groups, and *P*-values were calculated by Tukey’s test. Significance was determined at *P  < 0.033, **P  < 0.002 and ***P  < 0.001.

## Results

### Comparative sequence and phylogenetic analysis of *Hc*-CAP-15

**Fig. 1:**
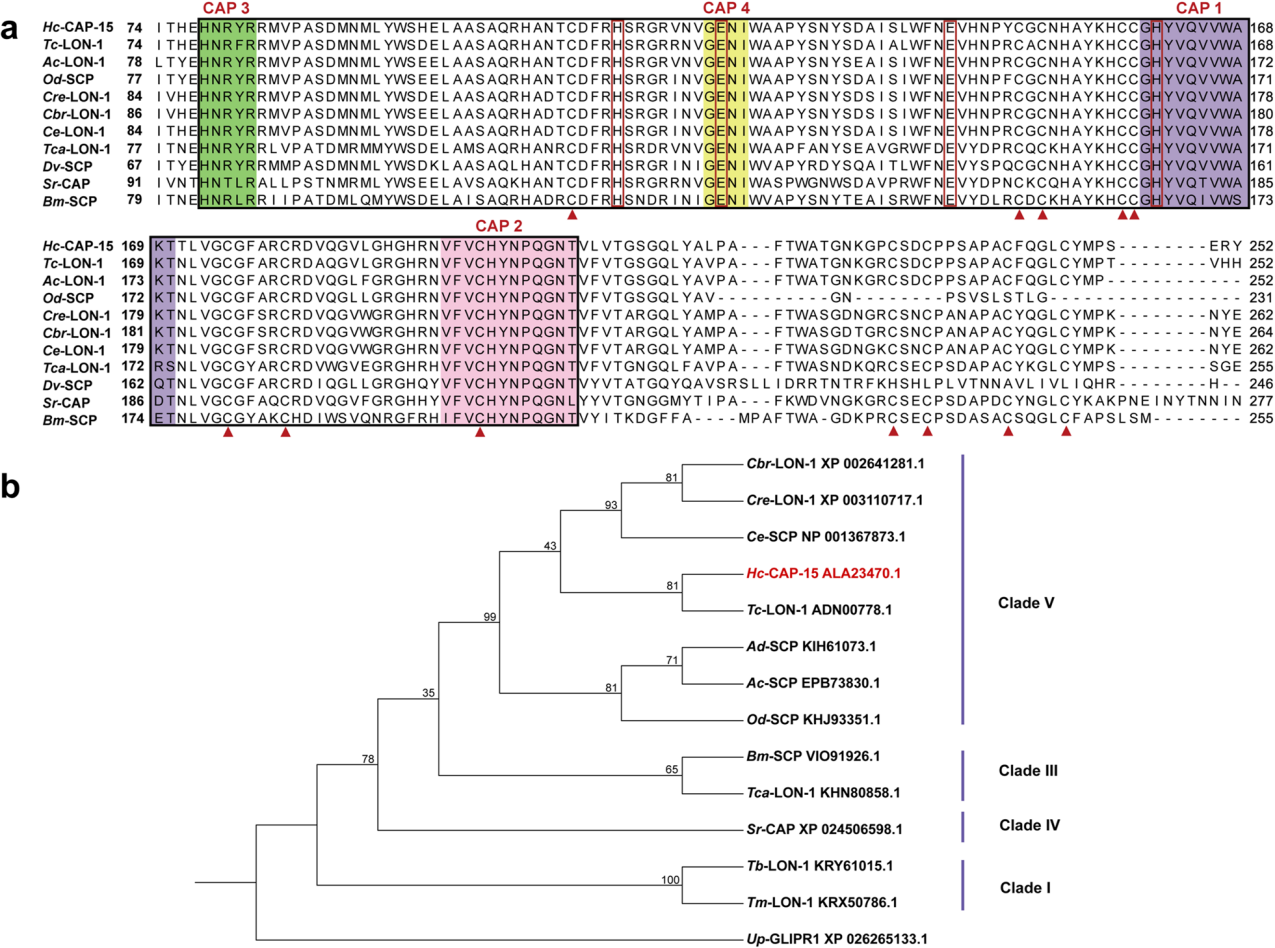
Multiple amino acid sequence alignment and phylogenetic analysis of *Hc*-CAP-15. **a** Sequence alignment of *Hc*-CAP-15 of *Haemonchus contortus* with 10 homologs of other nematode species. The CAP domain (Pfam database: pFam00188) is delineated by a black box. The CAP1 motif [(GDER) (HR) (FYWH) (TVS) (QA) (LIVM) (LIVMA) Wxx (STN)] is highlighted in purple, the CAP2 motif [(LIVMFYH) (LIVMFY) xC (NQRHS) Yx (PARH) x (GL) N (LIVMFYWDN)] is highlighted in pink, the CAP3 motif [(H) (N) xx (R)] is highlighted in green and the CAP4 motif [(G) (EQ) (N) (ILV)] is highlighted in yellow. The red triangles represent conserved cysteine residues, and the red box marks the amino acid residues constituting the CAP tetrad. **b** The rooted neighbor-joining tree showing the phylogenetic relationships of *Hc*-CAP-15 of *H*. *contortus* with 12 homologs of other species. The nodal support values (%) are shown above the branches

The coding sequence (CDS) of *Hc*-*cap*-*15* was 894 bp in length (GenBank: KT361571.1) and encoded a protein (*Hc*-CAP-15) of 298 amino acids. The amino acid sequence of *Hc*-CAP-15 was highly similar (55.4–77.7%) to homologs of other nematodes which had been annotated as CAP/SCP protein or LON protein (Additional file 1: Table S1). Multiple sequence alignment revealed that *Hc*-CAP-15 and its homologs possessed a single CAP domain comprising four conserved CAP motifs (CAP1-CAP4) (Fig. 1a). In addition, the cysteine residues associated with disulfide bond formation, such as Cys110, Cys149, Cys151, Cys158, Cys159, Cys175 and Cys180, were highly conserved. Also, the putative CAP cavity of *Hc*-CAP-15 was characterized by a tetrad of residues consisting of His114, Glu123, Glu143 and His161, which were the potential active sites of the CAP domain proposed in the structural study [35]. Phylogenetic analysis showed that *Hc*-CAP-15 clustered on the same branch as *Tc*-LON-1 (GenBank: ADN00778.1) of *Teladorsagia circumcincta* from nematode clade V (81% match) and that, overall, the phylogenetic relationship among *Hc*-CAP-15 and homologs was consistent with the phylogeny of nematodes (Fig. 1b).

### Expression profiles of *Hc*‑CAP‑15 in adult *H. contortus*

**Fig. 2:**
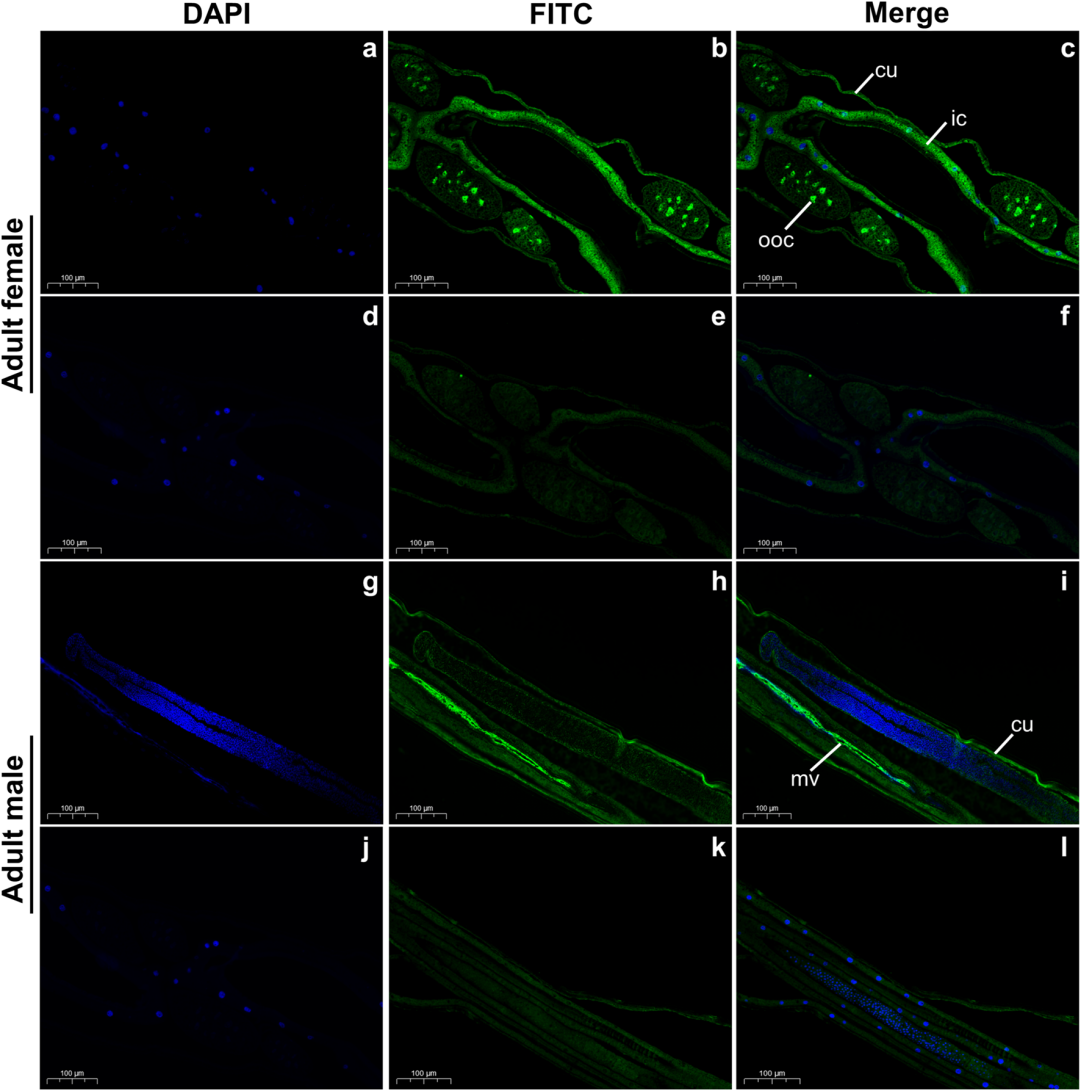
Tissue localization of *Hc*-CAP-15 in adult *Haemonchus contortus.* **a–c**, **g–i** Purified anti-r*Hc*-CAP-15 polyclonal antibody at a dilution of 1:500, **d–f**, **j–l** purified negative serum at an antibody dilution of 1:500. Scale bar: 100 μm. DAPI, 4,6-Diamidino-2-phenylindole; FITC, fluorescein isothiocyanate; cu, cuticle; ic, intestinal cytoplasm; mv, intestinal microvillus; ooc, oocyte in the ovary

The truncated recombinant (r) *Hc*-CAP-15 protein with an expected size of 45 kDa (containing a SUMO fusion protein and 6 × His-tag) was successfully expressed in *E. coli* and purified (Additional file 2: Figure S1a). Anti-r*Hc*-CAP-15 polyclonal antibody obtained from rabbits immunized with r*Hc*-CAP-15 specifically recognized the native *Hc*-CAP-15 (approx. 33 kDa) of the adult worm, as shown in Additional file 2: Figure S1b. To investigate the anatomical expression of *Hc*-CAP-15 in adult worms, we performed indirect immunofluorescence analysis, which revealed the expression of *Hc*-CAP-15 in the cuticle, intestinal cytoplasm and oocyte in the ovary of female adults (Fig. 2a–f), as well as in the cuticle and intestinal microvilli of male adults (Fig. 2g–l).

### Transcription profiles of *Hc-cap-15* throughout the developmental stages of* H. contortus*

**Fig. 3:**
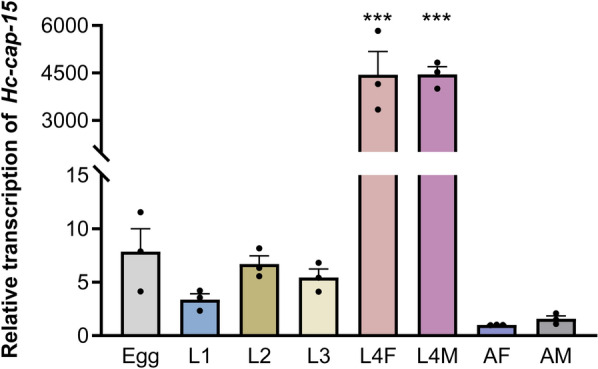
Transcriptional levels of *Hc-cap*-*15* at different developmental stages of *Haemonchus contortus.* Transcript abundance was detected and compared among eight developmental stages including eggs, first-stage larvae (L1s), second-stage larvae (L2s), third-stage larvae (L3s), fourth-stage female larvae (L4F), fourth-stage male larvae (L4M), adult female (AF) and adult male (AM) of *H. contortus.* The relative quantities (compared with AF, where AF = 1) are shown as the mean values (± standard error of the mean [SEM]) derived from three replicates in repeat experiments. All gene transcription levels were normalized to that of the *Hc*-*β-tubulin*. Asterisks indicate a significant difference between the stages at ***P < 0.001

Transcription was explored in eight stages/sexes of *H. contortus*, including eggs, L1, L2, L3, L4F, L4M, AF and AM. Real-time PCR analysis revealed that *Hc-cap*-*15* was transcribed at all crucial developmental periods. Interestingly, the transcription levels of this gene in both female and male L4s were significantly higher than that in any other stages (see Fig. 3), and there was no significant difference in the transcription levels between L4F and L4M.

### Assessment of the effect of *Hc-cap-15*-specific siRNA on larval development and growth of* H. contortus*

**Fig. 4:**
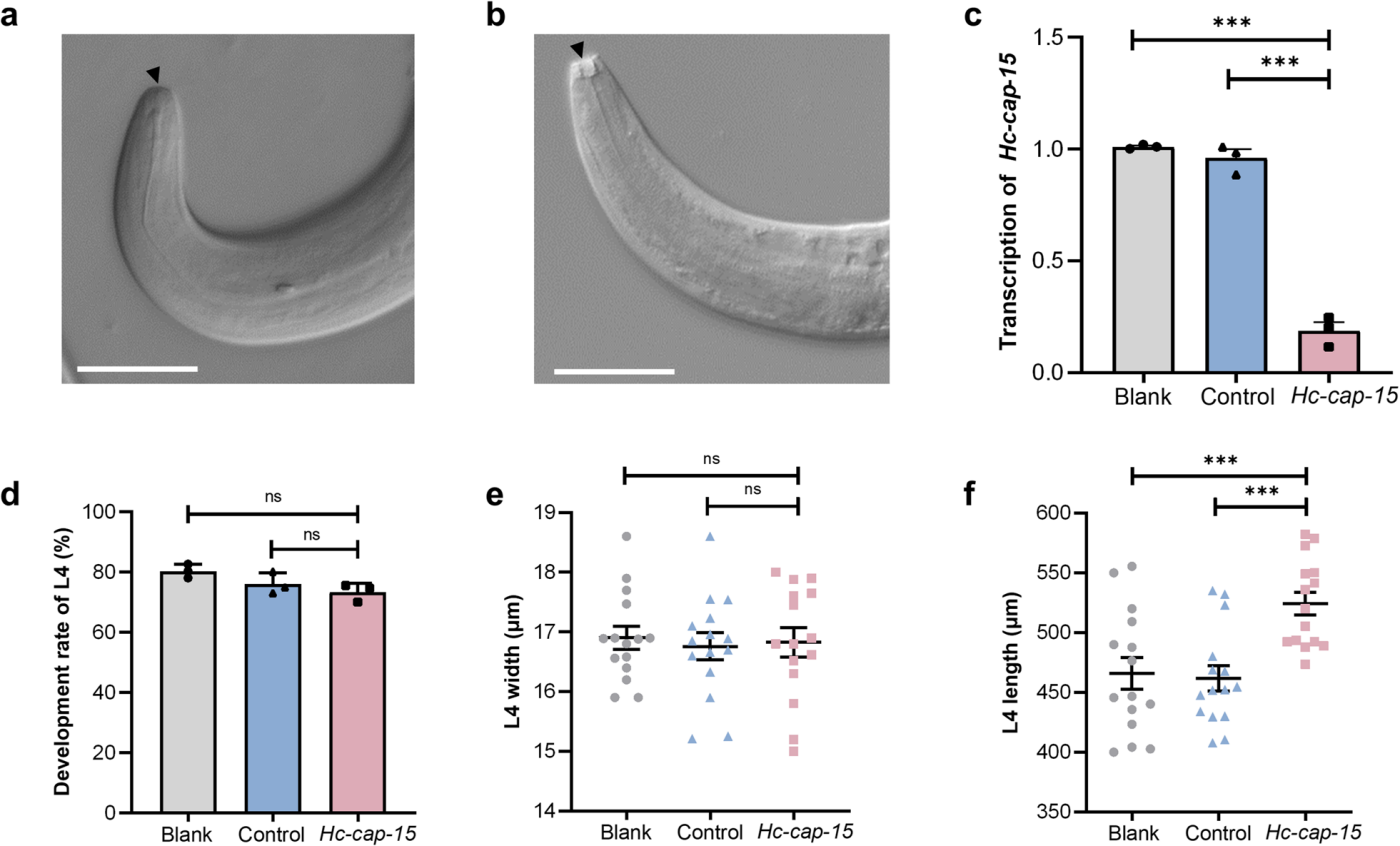
Effects of *Hc-cap-15* siRNA soaking treatment on *Haemonchus contortus* larval development. **a** Morphology of the mouth capsule of exsheathed L3 (xL3). **b** Morphology of the mouth capsule of L4s. **c** *Hc-cap-15* transcription abundance of *H. contortus* larvae after 96 h of RNAi by real-time PCR. **d** Developmental rate (%) of L4s (*n* = 100) on day 7 after RNAi. **e** Body width of *Hc-cap-15* siRNA-treated L4s (*n* = 15). **f** Body length of *Hc-cap-15* siRNA-treated L4s (*n* = 15). Scale bar: 30 μm. Asterisks indicate a significant difference between groups at ***P < 0.001; ns, no significance. L4s, Fourth-stage larvae; siRNA, small interfering RNA

Real-time PCR analysis showed a significant decrease in the transcription levels of *Hc-cap-15* following the exposure of xL3s to *Hc-cap-15* siRNAs in vitro using a soaking method, compared with the no-siRNA group (blank) and irrelevant-siRNA group (control) (Fig. 4a). During the development of xL3s to L4s of *H. contortus *in vitro, the xL3s showed a closed mouth (Fig. 4b) whereas the L4s showed an opened mouth (Fig. 4c). However, the downregulation of *Hc-cap-15* had no significant effect on the developmental rate of the larvae from the xL3s to the L4s (Fig. 4d) and the body width of L4s (Fig. 4e), but it did significantly increase the body length of L4s (Fig. 4f).

## Discussion

CAP proteins have been described to be ubiquitously present in various eukaryotic organisms and implicated in a range of functions, including sperm maturation in mammals, immune defense in plants and fungal virulence [36]. Despite their abundant occurrence in helminth genomes, very little is known about their biological function in parasitic nematodes. In the present study, we characterized *Hc*-CAP-15 as a single-domain CAP protein of *H. contortus* that was widely located in tissues of adult worms, such as the cuticle, intestine and oocyte of the ovary. Moreover, we determined that the highest transcription of *Hc-cap*-*15* occurred in parasitic L4s and that it was an important developmental regulatory molecule of *H. contortus*.

All members of the CAP protein superfamily share a common CAP domain which adopts a distinctive α–β–α sandwich folding pattern, with each loop stabilized by hydrophobic interactions, hydrogen bonds and disulfide bonds [35, 37–40]. In our study, *Hc*-CAP-15 was identified as a single-domain CAP protein containing four highly conserved CAP motifs (CAP1–CAP4) and 12 cysteine residues involved in disulfide bond formation. These features are thought to be related to the high thermal and proteolytic stability of CAP proteins, which corresponds to the structural requirements for extracellular functions [41, 42]. In addition, the four acid residues (two histidine and two glutamine) forming the "central cavity" were conserved in *Hc*-CAP-15, leading us to speculate that this protein has a potential active site for binding divalent cations, such as Zn^2+^ and Mg^2+^ [43–45]. Research on the crystal structure of CAP protein in *Brugia malayi* revealed that the central CAP cavity is connected to other cavities by channels that could serve as pathways for water molecules, cations and small molecules [35]. Hence, it is imperative to carry out further investigations into the three-dimensional structure of *Hc*-CAP-15 in order to unravel the relationship between structure and function.

The localization of *Hc*-CAP-15 in the worm also provided evidence for its functional prediction. We discovered that *Hc*-CAP-15 was expressed in the exterior cuticle of the adult nematode, a finding that is consistent with the localization of *Ce*-LON-1, a homolog in *Caenorhabditis elegans* [46]. This finding implies that *Hc*-CAP-15 could play a significant role in essential physiological processes, including endocrine signaling, fat storage and ion homeostasis, throughout nematode development [47]. *Hc*-CAP-15 was also detected in the intestinal cytoplasm of female adults and intestinal microvillus of male adults, indicating that its possible functions in blood-sucking nematodes are more complex than those of free-living nematodes, such as absorbing nutrients and regulating development. Moreover, many antigens distributed in the intestines are promising candidates for effective vaccines [48–51]. Interestingly, in adult *H. contortus*, *Hc*-CAP-15 was not observed in the male reproductive organs, but it was found to be abundant in the oocytes of ovaries in the female, suggesting that this protein is integral to egg production and/or pre-embryonic development and that its function differs significantly between males and females. It is worth noting that several CAP proteins have been localized in the pharyngeal and esophageal glands of some helminth species (such as* A. caninum* [52], *Onchocerca volvulus* [53] and *Schistosoma manson* [54]); however, we did not find *Hc*-CAP-15 in these tissues in our study.

In the present study, the highest transcription abundance of *Hc-cap-15* was determined at the parasitic L4s. As previously described, the parasitic L4 of *H. contortus* is a key period during the transition from the free-living to the parasitic phase due to the dramatic change in the living environment [14]. Our findings imply that *Hc-cap-15* could play a crucial role in acquiring nutrients and maintaining body shape as well as in parasite-host interactions in parasitic L4s. In *H. contortus*, two CAP proteins (Hc24 and Hc40) were previously found in excretory/secretory (ES) products of parasitic worms; the former could be recognized by the high levels of immune serum [55]. Moreover, the recombinant Hc24 protein could bind to goat peripheral blood mononuclear cells (PBMCs) and increase the production of interleukin (IL)-4, IL-10 and IL-17, and cell migration in vitro [56]. Therefore, a better understanding of the role of *Hc*-CAP-15 in immune regulation will provide new insights into the mechanisms of parasite-host interplay and identify new intervention targets.

Previous studies predicted that *Hc*-CAP-15 and *Ce*-LON-1 of *C. elegans* might play similar roles in regulating development based on their homology [29]. Abnormally long knockout mutants of the *Ce*-*lon*-*1* in *C. elegans* suggest a negative role in body size regulation [46, 57]. We proved that efficient knockdown of *Hc-cap*-*15* significantly increased the body length of L4s in vitro. This finding is consistent with the function of *Ce*-LON-1, indicating that *Hc*-CAP-15 is an important regulatory factor in the development progress of *H. contortus*. Transforming growth factor-beta (TGF-β) signaling is required during development and homeostasis for several vital life processes of nematodes, including the DBL-1, DAF-7 and UNC-129 signaling pathways [58]. In *C. elegans*, *Ce-lon-1* has been shown to interact with various genes up- or down-stream of the TGF-β signaling pathway, such as *Ce-dbl-1*, *Ce-sma-2*, *Ce-sma-3*, *Ce-sma-4*, *Ce-daf-4*, among others [57, 58]. Moreover, although information on the TGF-β signaling pathway in *H. contortus* is not yet fully defined, the genes related to the DAF-7 signaling pathway have been confirmed to have highly conserved molecular structure and function with *C. elegans* [59]*.* Based on the similar biological characteristics of *H. contortus* and *C. elegans* and the homology of the *Ce*-LON-1 and *Hc*-CAP-15, we predicted that the negative regulation of *Hc*-CAP-15 on body length may be the result of interaction with molecules involved in the TGF-β signaling pathway of *H. contortus*.

## Conclusion

In this study, we showed the *Hc*-CAP-15 of *H. contortus* with conserved sequence characteristics and phylogenetic relationships and suggested that this molecule plays a crucial role in regulating the development of parasitic L4s. These findings lay the foundation for future functional and mechanistic studies on CAP molecules, which will improve current understanding of the biology of *H. contortus* and other related parasitic nematodes.

### Supplementary Information

**Additional file 1: Table S1.** GenBank accession numbers of *Hc*-CAP-15 and its homologs from 13 nematode species and one non-nematode species used for alignment and phylogenetic analysis.** Table S2.** Information on oligonucleotide primers (5′-3′) used in the present study.** Table S3.** Information on *Hc-cap-15*-specific siRNAs and control siRNA used in the RNA interference assay.

**Additional file 2: Figure S1.** Prokaryotic expression of recombinant *Hc*-CAP-15 protein and immunoblot analysis. **a** Sodium dodecyl sulfate–polyacrylamide gel electrophoresis (SDS-PAGE) of expression and purification of recombinant Hc-CAP-15 protein.* Lanes*:* M*, Protein ladder;* 1*, induced empty pE-SUMO expression vector by 1 mM isopropyl β-D-1-thiogalactopyranoside (IPTG) at 37 °C;* 2*, un-induced r*Hc*-CAP-15;* 3*, induced r*Hc*-CAP-15 by 1 mM IPTG at 37 °C;* 4*, supernatant of r*Hc*-CAP-15;* 5*, inclusion body of r*Hc*-CAP-15;* 6*, outflow liquid;* 7*, purified r*Hc*-CAP-15. **b** Immunoblot of IgG antibodies from rabbit serum binding to native *Hc*-CAP-15 protein.* Lanes*:* 1*, Positive IgG antibody;* 2*, negative IgG antibody.

## Data Availability

Data supporting the conclusions of this article are included within the article and its additional files.

## Abbreviations

BSA: Bovine serum albumin
DAPI: 4,6-Diamidino-2-phenylindole
EBSS: Earle’s balanced salt solution
FITC: Fluorescein isothiocyanate
iL3s: Infective third-stage larvae
IPTG: Isopropyl β-D-1-thiogalactopyranoside
L4s: Fourth-stage larvae
PBS: Phosphate-buffered saline
RNAi: RNA interference
SDS-PAGE: Sodium dodecyl sulfate–polyacrylamide gel electrophoresis
siRNA: Small interfering RNA
TGF-β: Transforming growth factor beta
xL3s: Exsheathed third-stage larvae
