# Supplementary material for: A member of the CAP protein superfamily, Hc-CAP-15, is important for the parasitic-stage development of Haemonchus contortus
Source: Parasit Vectors. 2023 Aug 17;16:290. doi: 10.1186/s13071-023-05907-w (PMC10433639; doi:10.1186/s13071-023-05907-w)
Supplement: Supplementary file 1 — Additional file 1: Table S1. GenBank accession numbers of Hc-CAP-15 and its homologs from 13 nematode species and one non-nematode species used for alignment and phylogenetic analysis. Table S2. Information on oligonucleotide primers (5′-3′) used in the present study. Table S3. Information on Hc-cap-15-specific siRNAs and control siRNA used in the RNA interference assay. [file 13071_2023_5907_MOESM1_ESM.docx]

**Table S1.** GenBank accession numbers of *Hc*-CAP-15 and its homologs from 13 nematode species and one non-nematode species used for alignment and phylogenetic analysis.

| **Species** | **GenBank number** | **Protein annotation** |
| --- | --- | --- |
| *Ancylostoma duodenale* | KIH61073.1 ^b^ | SCP-like protein |
| *Ancylostoma ceylanicum* | EPB73830.1 ^a^ ^b^ | SCP-like protein |
| *Brugia malayi* | VIO91926.1 ^a^ ^b^ | SCP-like extracellular protein |
| *Caenorhabditis briggsae* | XP_002641281.1 ^a^ ^b^ | LON-1 protein |
| *Caenorhabditis elegans* | NP_001367873.1 ^a^ ^b^ | SCP domain-containing protein |
| *Caenorhabditis remanei* | XP_003110717.1 ^a b^ | LON-1 protein |
| *Dictyocaulus viviparus* | KJH53182.1 ^a^ | SCP-like protein |
| *Haemonchus contortus* | ALA23470.1 ^a b^ | *Hc*-CAP-15 |
| *Oesophagostomum dentatum* | KHJ93351.1 ^a b^ | SCP-like protein |
| *Strongyloides ratti* | XP_024506598.1 ^a^ ^b^ | CAP domain-containing protein |
| *Teladorsagia circumcincta* | ADN00778.1 ^a^ ^b^ | *Tc*-LON-1 |
| *Toxocara canis* | KHN80858.1 ^a^ ^b^ | *Tca*-LON-1 |
| *Trichinella britovi* | KRY61015.1 ^b^ | *Tb*- LON-1 |
| *Trichinella murrelli* | KRX50786.1 ^b^ | *Tm*-LON-1 |
| *Urocitellus parryii* | XP_026265133.1 ^c^ | GLIPR1-like protein 1 |

^a^ Sequence used for amino acid sequence alignment; ^b^ Sequence used to construct phylogenetic tree; ^c^ Peripheral root sequence used to construct phylogenetic tree

**Table S2.** Information on oligonucleotide primers (5’-3’) used in the present study.

| **Primer** | **Sequence (5’-3’)** |
| --- | --- |
| *Hc-cap-15* amplification | |
| *Hc-cap-15*-F | ATGCACTCTTCACTGCTTCTC |
| *Hc-cap*-*15*-R | TCAGAAACCTGGGTCGGAC |
| Transcription level analysis | |
| *qHc-cap-15*-F | ATTGTGTTGCAGTGGTGTGC |
| q*Hc-cap*-*15*-R | CAGAACGCTTGACTTGACCG |
| *qHc*-*tubulin*-F | TGTTCCATCACCCAAGGTATCC |
| *qHc*-*tubulin*-R | TGACAGACACAAGGTGGTTGAGAT |
| Prokaryotic expression of *Hc*-CAP-15 | |
| r*Hc-*CAP-15-F | ACCGCGAACAGATTGGAGGTGGCGCACAAATATCGTAT |
| r*Hc*-CAP-15-R | TCGAATTCGGATCCTCTAGTTCAGAAACCTGGGTCGGAC |

**Table S3.** Information on *Hc-cap-15*-specific siRNAs and control siRNA used in the RNA interference assay.

| **Name** | **Sequence** |
| --- | --- |
| siRNA-1 | Sense: 5’- GGAUUACUCAUGAGCACAATT’ |
|  | anti-sense: 5’- UUGUGCUCAUGAGUAAUCCTT -3’ |
| siRNA-2 | Sense: 5’- GCAGCUCCCUACAGCAAUUTT-3’ |
|  | anti-sense: 5’- AAUUGCUGUAGGGAGCUGCTT -3’ |
| siRNA-3 | Sense: 5’- CCAUUUCACUAUGGUUCAATT-3’ |
|  | anti-sense: 5’- UUGAACCAUAGUGAAAUGGTT -3’ |
| Control siRNA | Sense: 5’-UUCUCCGAACGUGUCACGUTT-3’ |
|  | anti-sense: 5’-ACGUGACACGUUCGGAGAATT-3’ |
