# Supplementary figures and images for: A member of the CAP protein superfamily, Hc-CAP-15, is important for the parasitic-stage development of Haemonchus contortus
Source: Parasit Vectors. 2023 Aug 17;16:290. doi: 10.1186/s13071-023-05907-w (PMC10433639; doi:10.1186/s13071-023-05907-w)

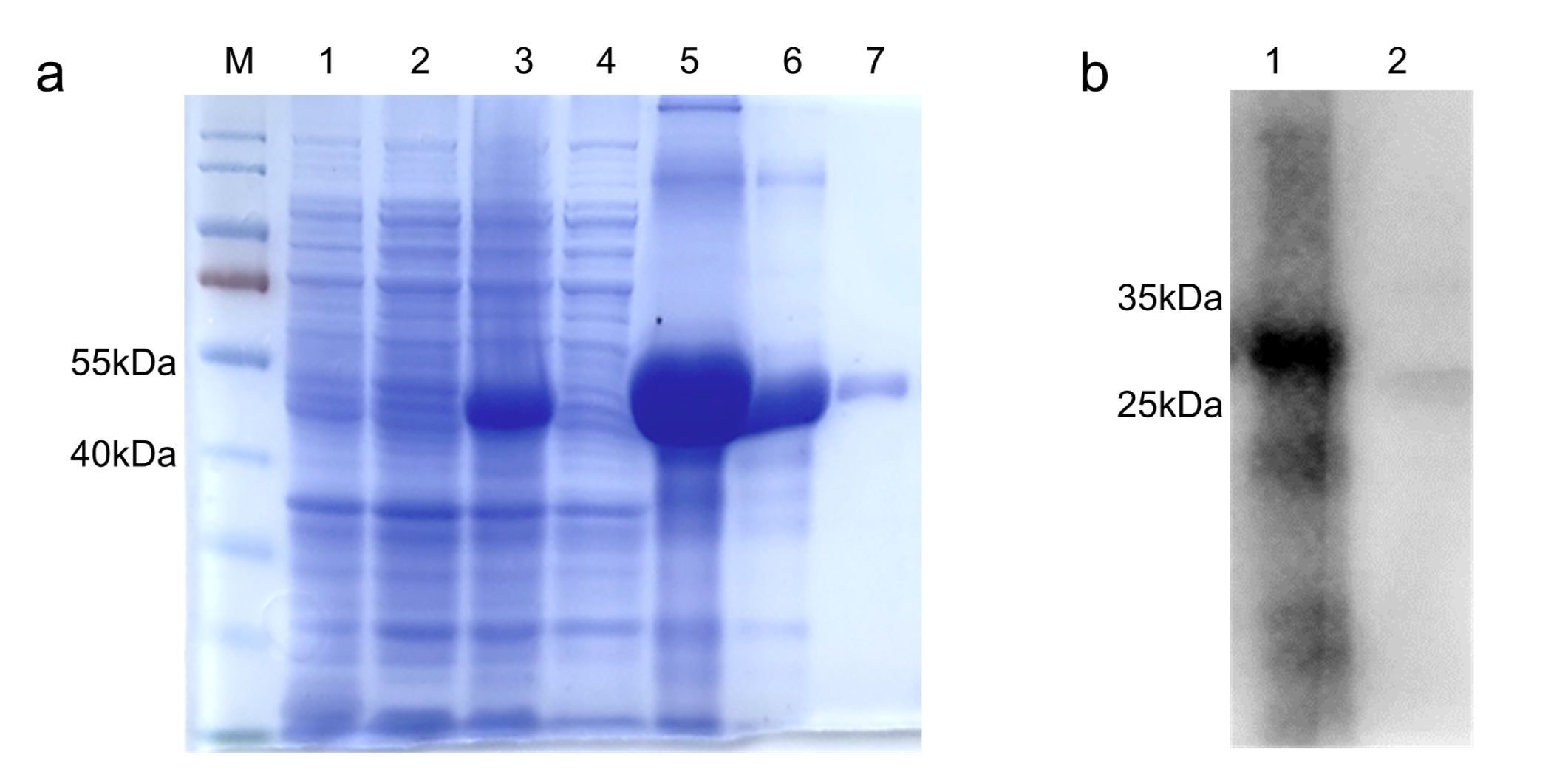

Supplement: Supplementary file 2 — Additional file 2: Figure S1. Prokaryotic expression of recombinant Hc-CAP-15 protein and immunoblot analysis. a Sodium dodecyl sulfate–polyacrylamide gel electrophoresis (SDS-PAGE) of expression and purification of recombinant Hc-CAP-15 protein. Lanes: M, Protein ladder; 1, induced empty pE-SUMO expression vector by 1 mM isopropyl β-D-1-thiogalactopyranoside (IPTG) at 37 °C; 2, un-induced rHc-CAP-15; 3, induced rHc-CAP-15 by 1 mM IPTG at 37 °C; 4, supernatant of rHc-CAP-15; 5, inclusion body of rHc-CAP-15; 6, outflow liquid; 7, purified rHc-CAP-15. b Immunoblot of IgG antibodies from rabbit serum binding to native Hc-CAP-15 protein. Lanes: 1, Positive IgG antibody; 2, negative IgG antibody. [file 13071_2023_5907_MOESM2_ESM.tif]
